# Supplementary material for: MiR-199a-3p Induces Mesenchymal to Epithelial Transition of Keratinocytes by Targeting RAP2B
Source: Int J Mol Sci. 2022 Dec 6;23(23):15401. doi: 10.3390/ijms232315401 (PMC9741271; doi:10.3390/ijms232315401)
Supplement: Supplementary file 1 [file ijms-23-15401-s001.zip › ijms-1895329-supplementary.pdf]

Supplementary data

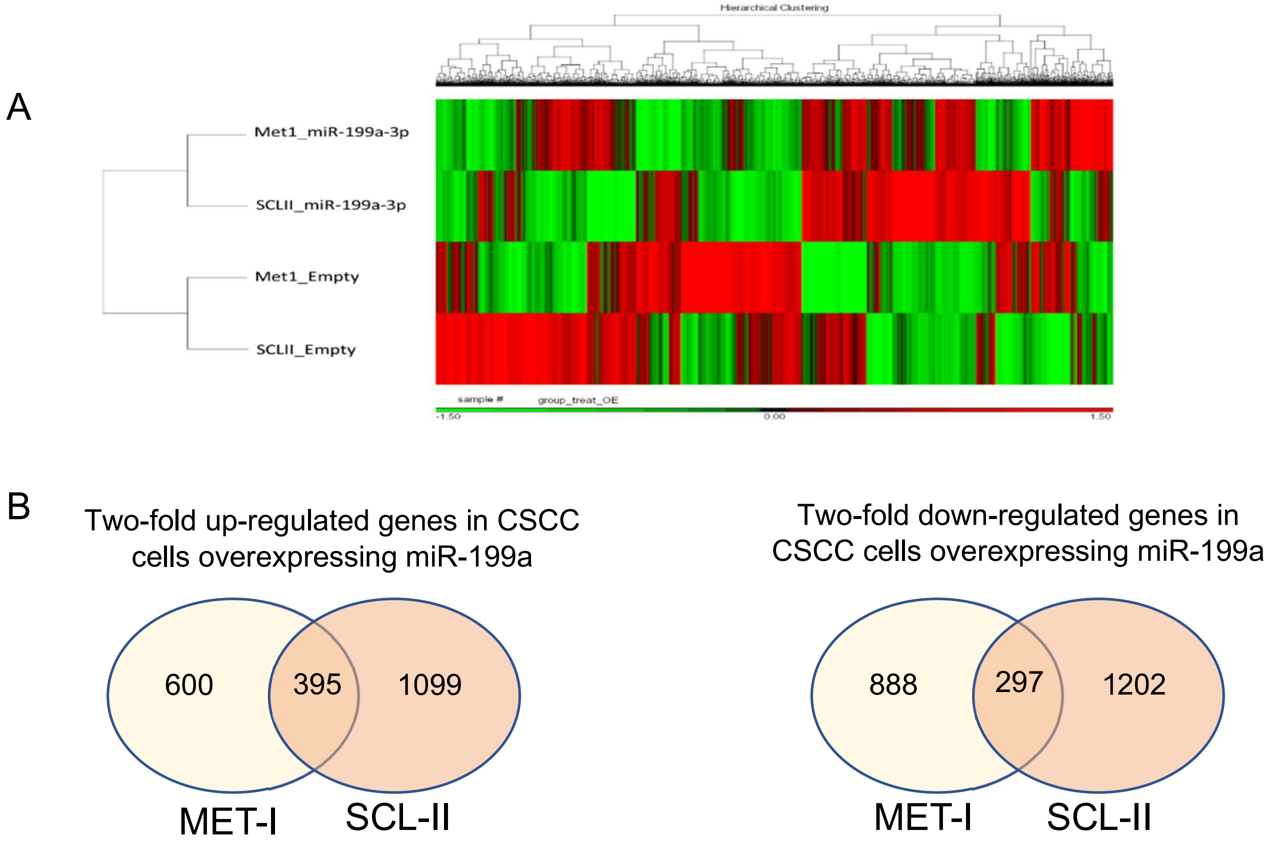

Figure S1: mRNA expression profiling in miR-199-overexpressing and control cells. (**A**) Heatmap and hierarchical clustering using Partek Genomic suite 6.6 Spotfire Decision Site for Functional Genomics (Somerville). (**B**) Venn diagrams present a summary of the number of genes changed at least 2-fold in cells overexpressing miR-199a-3p compared to control cells.

Table S1: effect of miR-199a expression on EMT-related mRNA genes expression found in Affymetrix mRNA array (the number presented as fold change are (-1)x1/miR-199a-3p/control)

Gene Symbol	Fold Change MET1 (miR-199a vs. empty)	Fold Change SCL-II (miR-199a vs. empty)
SMAD4	-5.5	-10.6
SERPINE1	-2.3	-1.9
SERPINE2	-3.10	-6.10
ID1	-5.17	-12.72
ID2	-9.83	-23.31
COL4A1	-2.39	-2.9
VIM	-237.50	-1.30
SMAD3	-2.63	-1.12

Table S2: Genes found to decrease in both CSCC cells by at least two folds and predicted to contain putative miR-199a-3p binding site by at least 4 out of 12 different bioinformatics programs (the number presented as fold change are (-1)x1/miR-199a-3p/control)

Gene Symbol	Association with EMT, proliferation or migration of cells	Fold Change MET1 (miR-199a vs. empty)	Fold Change SCL-II (miR-199a vs. empty)	Number of programs out of 12 that predicted a target of miR-199a-3p
ABLIM3	Component of adherence junctions [1]	-6.32	-3.75	5
AGPAT9	Suppresses cell growth, invasion and metastasis [2]	-3.05	-2.35	7
AKAP2	Promotes growth and migration [3]	-3.05	-2.09	5
ALDH1A3	Plays a role in EMT [4]	-2.39	-2.26	5
AMIGO2	Affects migration and tumorigenicity [5]	-6.74	-3.56	5
AXL	Promotes invasiveness-metastasis, and EMT phenotype [6]	-6.88	-4.26	4
BCL2L13		-2.88	-2.33	8
CHST11	Promotes EMT [7]	-6.97	-6.56	5
COL4A6		-2.61	-2.50	4
CYP1B1	Enhances cell proliferation and metastasis through induction of EMT [8]	-10.98	-2.245	8
DIO2		-3.56	-4.28	9
DLX2	Induction of EMT [9]	-3.03	-2.92	6
DSEL		-5.73	-2.09	7
DUSP5	Suppresses proliferation, invasion, and migration [10]	-2.12	-2.67	9
FAM198B	Correlated with signatures of "EMT", "metastasis," and "invasion" [11]	-3.16	-2.36	6
FGF2	Promotes EMT [12]	-2.96	-2.11	6
FN1	Promotes EMT [13]	-2.52	-6.22	9
FRMD5	Regulates tumor cell motility [14]	-2.43	-2.11	4
GALNT18		-2.11	-5.32	6
GRK5	Promotes tumor progression [15]	-4.31	-2.67	4
GTF2H1 (P62)		-2.30	-2.28	4
HMCN1	Enhances migration and invasion [16]	-3.70	-2.03	4
ID2	Promotes EMT [17]	-10.18	-23.75	6
JAG1 (Jagged1)	Promotes EMT[18]	-3.76	-2.20	4
LAMC2	promotes EMT[19]	-2.10	-2.07	6
LIF	promotes EMT [20]	-4.18	-2.97	4
MALL		-2.09	-2.74	6
MPRIP		-2.02	-2.37	4
NRG1	Promotes EMT [21]	-4.90	-2.40	4

NRIP1 (RIP140)	Promotes growth and proliferation [22]	-2.13	-2.38	6
NRP1	Promotes EMT [23]	-4.37	-2.85	6
NRP2	Promotes EMT [24]	-2.06	-2.42	5
NTRK2	Promotes EMT [25]	-3.35	-2.27	6
PHLDA1 (TDAG51)	Promotes EMT [26]	-3.99	-7.98	4
PPP4R4		-6.47	-4.21	4
PVRL3		-9.22	-2.36	6
PYGB	Promotes cell proliferation and migration [27]	-2.17	-2.31	5
RPS6KA2		-9.81	-3.72	5
RRAS2	Promotes proliferation and migration [28]	-2.78	-3.69	4
SERPINE2	Promotes EMT [29]	-3.09	-7.77	11
SH3KBP1 (CIN85)	Promotes proliferation [30]	-2.26	-5.97	5
SLC20A2		-13.94	-20.66	7
SMAD4	Promotes EMT [31]	-3.00	-4.50	4
TENM3		-10.98	-3.91	4
TRAF3	Inhibits proliferation, migration, and invasion [32]	-2.22	-2.59	5
TRIM31	Promotes proliferation, invasion and migration [33]	-2.40	-8.69	4
UBASH3B	Promotes invasion and metastasis [34]	-3.19	-2.52	5
USP36	Increase cell proliferation and malignant cell transformation [35]	-2.05	-2.49	4
VNN1		-7.18	-2.66	7
ZDHHC14	Cell migration and invasion [36]	-2.40	-2.09	6

Table S3: mRNA 3'UTRs bound by Ago2 near a miR-199a-3p complimentary region

Gene	Association with proliferation, migration, or EMT	Fold-change in MET1 (mir-199a vs. empty)	Fold-change in SCL-II (mir-199a vs. empty)
AASDHPPT			
ADAR	EMT [37]		
AIM1			
AKT3			
AMIGO2		-5.6	-4.0
BIRC5	EMT [38]	-1.6	-2.3
CCNT1 (Cyclin T)	EMT [39]		
CDK17		-2.346	1.1
CHD7	Proliferation [40]		
CHORDC1		-1.1	-2.0
CHTF8			
CRYBG3			
CSRP2	Invasion and metastasis [41]		
DERL1 (derlin-1)	EMT [42]		
DPH5			
EMC1			
FAM49B			
FAM96A			
FARSA			
FOXQ1	EMT [43]		
GGA3	Migration [44]	-1.8	-0.7
GOLGA1			
GRHL3	Migration and invasion [45]		
HDGF	Growth, migration and invasion [46]	-0.4	-2.8
HIST1H2BK			
KIF5B	EMT [47]	-1.3	-2.2
LDOC1L			
MESDC1			
MSN (moesin)	EMT [48]		
NACC2		-1.3	-2.2
NDC1 (TMEM48)	Proliferation, migration and invasion [49]		
PNRC1		1.8	3.7
RAD9A			
RAP2B	Proliferation, migration, and invasion [50]		
RNF141		-1.0	-1.9
SLC38A2			
SYTL2	Metastatic potential [51]		
TFAP2A	EMT [52]		
TGFBR3 (type III TGF-β receptor)	EMT [53]		
THBS1	EMT [54]	-1.6	-3.5
TNS4	EMT [55]		
TRAF3		-2.2	-1.9
VANGL (KITENIN)	EMT [56]		
VIPAS39			
VPS33A			
YKT6			
YWHAE (14-3-3ε)	EMT [57]	1.4	-2.3
ZBTB25			
ZMYND11			

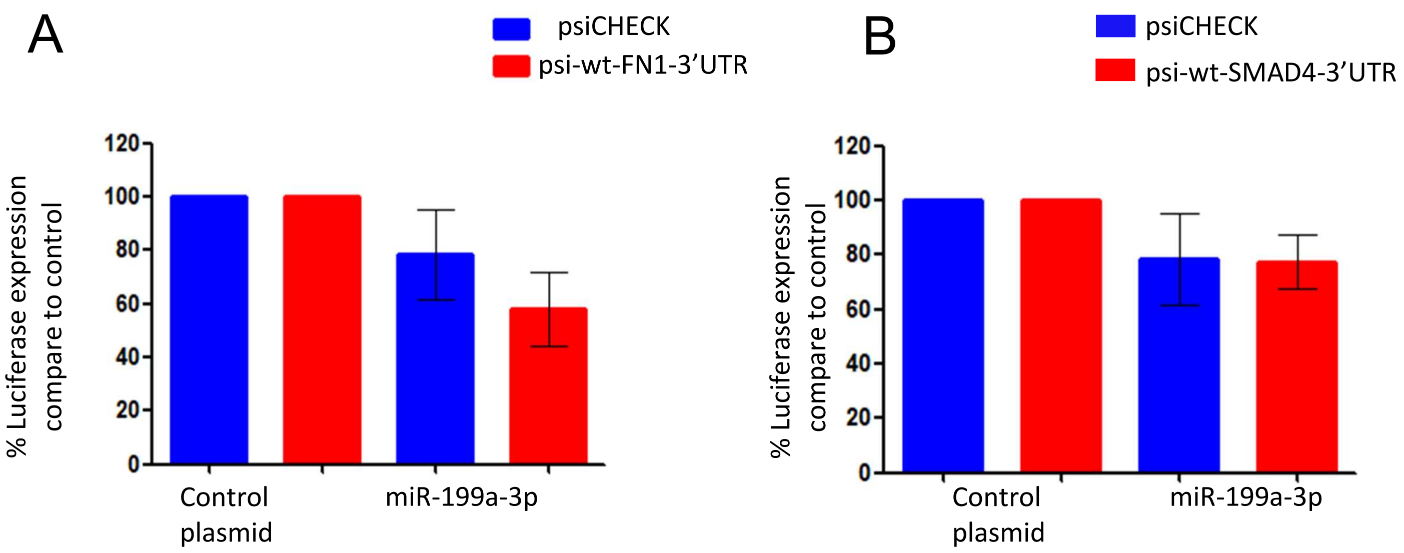

Figure S2: Effect of miR-199a on the 3'UTR of FN1 or SMAD4

SCL-II cells stably expressing miR-199a-3p were transfected with psiCHECK-II (control psiCHECK) or with a psiCHECK-II vector with WT 3'UTR of FN1 (A) or SMAD4 (B) fused to renilla luciferase (FN1 or SMAD4 columns respectively). The ratio of expression of renilla/firefly in cells transfected with psiCHECK-II (control) was set as 100% and the results are presented relative to it. (The mean ± SD was calculated from 5 independent experiments. Statistics were performed with t-tests. Both p-values of FN1 vs. control and SMAD4 vs, control were higher than 0.05.

Supplementary References

[1] Matsuda M, Yamashita JK, Tsukita S, Furuse M. abLIM3 is a novel component of adherens junctions with actin-binding activity. European Journal of cell biology. 2010;89; 807-816.

[2] Fan SH, Wang YY, Wu ZY, Zhang ZF, Lu J, Li MQ, et al. AGPAT9 suppresses cell growth, invasion and metastasis by counteracting acidic tumor microenvironment through KLF4/LASS2/V-ATPase signaling pathway in breast cancer. Oncotarget. 2015;6; 18406-18417.

[3] Li X, Wang C, Zhang G, Liang M, Zhang B. AKAP2 is upregulated in ovarian cancer, and promotes growth and migration of cancer cells. Mol Med Rep. 2017;16; 5151-5156.

[4] Yamashita D, Minata M, Ibrahim AN, Yamaguchi S, Coviello V, Bernstock JD, et al. Identification of ALDH1A3 as a Viable Therapeutic Target in Breast Cancer Metastasis-Initiating Cells. Molecular cancer therapeutics. 2020;19; 1134-1147.

[5] Rabenau KE, O'Toole JM, Bassi R, Kotanides H, Witte L, Ludwig DL, et al. DEGA/AMIGO-2, a leucine-rich repeat family member, differentially expressed in human gastric adenocarcinoma: effects on ploidy, chromosomal stability, cell adhesion/migration and tumorigenicity. Oncogene. 2004;23; 5056-5067.

[6] Wu X, Liu X, Koul S, Lee CY, Zhang Z, Halmos B. AXL kinase as a novel target for cancer therapy. Oncotarget. 2014;5; 9546-9563.

[7] Behrens A, Jousheghany F, Yao-Borengasser A, Siegel ER, Kieber-Emmons T, Monzavi-Karbassi B. Carbohydrate (Chondroitin 4) Sulfotransferase-11-Mediated Induction of Epithelial-Mesenchymal Transition and Generation of Cancer Stem Cells. Pharmacology. 2020;105; 246-259.

[8] Kwon YJ, Baek HS, Ye DJ, Shin S, Kim D, Chun YJ. CYP1B1 Enhances Cell Proliferation and Metastasis through Induction of EMT and Activation of Wnt/β-Catenin Signaling via Sp1 Upregulation. PLoS ONE. 2016;11; e0151598.

[9] Choi YJ, Baek GY, Park HR, Jo SK, Jung U. Smad2/3-Regulated Expression of DLX2 Is Associated with Radiation-Induced Epithelial-Mesenchymal Transition and Radioresistance of A549 and MDA-MB-231 Human Cancer Cell Lines. PLoS ONE. 2016;11; e0147343.

[10] Wang R, Bao HB, Du WZ, Chen XF, Liu HL, Han DY, et al. P68 RNA helicase promotes invasion of glioma cells through negatively regulating DUSP5. Cancer Sci. 2019;110; 107-117.
[11] Zhang Z, Liu R, Jin R, Fan Y, Li T, Shuai Y, et al. Integrating Clinical and Genetic Analysis of Perineural Invasion in Head and Neck Squamous Cell Carcinoma. Front Oncol. 2019;9; 434.
[12] Koike Y, Yozaki M, Utani A, Murota H. Fibroblast growth factor 2 accelerates the epithelial-mesenchymal transition in keratinocytes during wound healing process. Sci Rep. 2020;10; 18545.
[13] Li B, Shen W, Peng H, Li Y, Chen F, Zheng L, et al. Fibronectin 1 promotes melanoma proliferation and metastasis by inhibiting apoptosis and regulating EMT. Onco Targets Ther. 2019;12; 3207-3221.
[14] Hu J, Niu M, Li X, Lu D, Cui J, Xu W, et al. FERM domain-containing protein FRMD5 regulates cell motility via binding to integrin β5 subunit and ROCK1. FEBS letters. 2014;588; 4348-4356.
[15] Zhao TL, Gan XX, Bao Y, Wang WP, Liu B, Wang LH. GRK5 promotes tumor progression in renal cell carcinoma. Neoplasma. 2019;66; 261-270.
[16] Liu CL, Pan HW, Torng PL, Fan MH, Mao TL. SRPX and HMCN1 regulate cancer-associated fibroblasts to promote the invasiveness of ovarian carcinoma. Oncology reports. 2019;42; 2706-2715.
[17] Zhou JP, Gao ZL, Zhou ML, He MY, Xu XH, Tao DT, et al. Snail interacts with Id2 in the regulation of TNF-α-induced cancer cell invasion and migration in OSCC. Am J Cancer Res. 2015;5; 1680-1691.
[18] Shao S, Zhao X, Zhang X, Luo M, Zuo X, Huang S, et al. Notch1 signaling regulates the epithelial-mesenchymal transition and invasion of breast cancer in a Slug-dependent manner. Mol Cancer. 2015;14; 28.
[19] Pei YF, Liu J, Cheng J, Wu WD, Liu XQ. Silencing of LAMC2 Reverses Epithelial-Mesenchymal Transition and Inhibits Angiogenesis in Cholangiocarcinoma via Inactivation of the Epidermal Growth Factor Receptor Signaling Pathway. Am J Pathol. 2019;189; 1637-1653.
[20] Yue X, Zhao Y, Zhang C, Li J, Liu Z, Liu J, et al. Leukemia inhibitory factor promotes EMT through STAT3-dependent miR-21 induction. Oncotarget. 2016;7; 3777-3790.
[21] Shi DM, Li LX, Bian XY, Shi XJ, Lu LL, Zhou HX, et al. miR-296-5p suppresses EMT of hepatocellular carcinoma via attenuating NRG1/ERBB2/ERBB3 signaling. J Exp Clin Cancer Res. 2018;37; 294.
[22] Yu XH, Xue X, Zhu X, Li X. Downregulation of RIP140 in triple-negative breast cancer inhibits the growth and proliferation of cancer cells. Oncology letters. 2018;15; 8784-8788.
[23] Chu W, Song X, Yang X, Ma L, Zhu J, He M, et al. Neuropilin-1 promotes epithelial-to-mesenchymal transition by stimulating nuclear factor-kappa B and is associated with poor prognosis in human oral squamous cell carcinoma. PLoS ONE. 2014;9; e101931.
[24] Prud'homme GJ, Glinka Y. Neuropilins are multifunctional coreceptors involved in tumor initiation, growth, metastasis and immunity. Oncotarget. 2012;3; 921-939.
[25] Li Z, Zhou Z, Wu X, Zhou Q, Liao C, Liu Y, et al. LMP1 promotes nasopharyngeal carcinoma metastasis through NTRK2-mediated anoikis resistance. Am J Cancer Res. 2020;10; 2083-2099.
[26] Carlisle RE, Heffernan A, Brimble E, Liu L, Jerome D, Collins CA, et al. TDAG51 mediates epithelial-to-mesenchymal transition in human proximal tubular epithelium. American journal of physiology Renal physiology. 2012;303; F467-481.
[27] Zhan Y, Chen R, Wang T, Shan S, Zhu H. Glycogen phosphorylase B promotes cell proliferation and migration through PI3K/AKT pathway in non-small cell lung cancer. Experimental lung research. 2020; 1-10.
[28] Wang K, Peng K. RRAS2 knockdown suppresses osteosarcoma progression by inactivating the MEK/ERK signaling pathway. Anti-cancer drugs. 2019;30; 933-939.
[29] Zhang J, Luo A, Huang F, Gong T, Liu Z. SERPINE2 promotes esophageal squamous cell carcinoma metastasis by activating BMP4. Cancer Lett. 2020;469; 390-398.
[30] Wakasaki T, Masuda M, Niiro H, Jabbarzadeh-Tabrizi S, Noda K, Taniyama T, et al. A critical role of c-Cbl-interacting protein of 85 kDa in the development and progression of head and neck squamous cell carcinomas through the ras-ERK pathway. Neoplasia. 2010;12; 789-796.
[31] Vincent T, Neve EP, Johnson JR, Kukalev A, Rojo F, Albanell J, et al. A SNAIL1-SMAD3/4 transcriptional repressor complex promotes TGF-beta mediated epithelial-mesenchymal transition. Nature cell biology. 2009;11; 943-950.
[32] Hu W, Guo G, Chi Y, Li F. Construction of Traf3 knockout liver cancer cell line using CRISPR/Cas9 system. J Cell Biochem. 2019;120; 14908-14915.
[33] Shi G, Lv C, Yang Z, Qin T, Sun L, Pan P, et al. TRIM31 promotes proliferation, invasion and migration of glioma cells through Akt signaling pathway. Neoplasma. 2019;66; 727-735.
[34] Lee ST, Feng M, Wei Y, Li Z, Qiao Y, Guan P, et al. Protein tyrosine phosphatase UBASH3B is overexpressed in triple-negative breast cancer and promotes invasion and metastasis. Proc Natl Acad Sci U S A. 2013;110; 11121-11126.

[35] Kim SY, Choi J, Lee DH, Park JH, Hwang YJ, Baek KH. PME-1 is regulated by USP36 in ERK and Akt signaling pathways. FEBS letters. 2018;592; 1575-1588.
[36] Oo HZ, Sentani K, Sakamoto N, Anami K, Naito Y, Uraoka N, et al. Overexpression of ZDHHC14 promotes migration and invasion of scirrhous type gastric cancer. Oncology reports. 2014;32; 403-410.
[37] Liu X, Fu Y, Huang J, Wu M, Zhang Z, Xu R, et al. ADAR1 promotes the epithelial-to-mesenchymal transition and stem-like cell phenotype of oral cancer by facilitating oncogenic microRNA maturation. J Exp Clin Cancer Res. 2019;38; 315.
[38] Zhang P, Zhao G, Ji L, Yin J, Lu L, Li W, et al. Knockdown of survivin results in inhibition of epithelial to mesenchymal transition in retinal pigment epithelial cells by attenuating the TGFbeta pathway. Biochem Biophys Res Commun. 2018;498; 573-578.
[39] Ji X, Lu H, Zhou Q, Luo K. LARP7 suppresses P-TEFb activity to inhibit breast cancer progression and metastasis. Elife. 2014;3; e02907.
[40] Ohta S, Yaguchi T, Okuno H, Chneiweiss H, Kawakami Y, Okano H. CHD7 promotes proliferation of neural stem cells mediated by MIF. Mol Brain. 2016;9; 96.
[41] Hoffmann C, Mao X, Dieterle M, Moreau F, Al Absi A, Steinmetz A, et al. CRP2, a new invadopodia actin bundling factor critically promotes breast cancer cell invasion and metastasis. Oncotarget. 2016;7; 13688-13705.
[42] Wu Z, Wang C, Zhang Z, Liu W, Xu H, Wang H, et al. High Expression of Derlin-1 Is Associated with the Malignancy of Bladder Cancer in a Chinese Han Population. PLoS ONE. 2016;11; e0168351.
[43] Fan DM, Feng XS, Qi PW, Chen YW. Forkhead factor FOXQ1 promotes TGF-beta1 expression and induces epithelial-mesenchymal transition. Molecular and cellular biochemistry. 2014;397; 179-186.
[44] Ratcliffe CD, Sahgal P, Parachoniak CA, Ivaska J, Park M. Regulation of Cell Migration and beta1 Integrin Trafficking by the Endosomal Adaptor GGA3. Traffic. 2016;17; 670-688.
[45] Zhao P, Guo S, Tu Z, Di L, Zha X, Zhou H, et al. Grhl3 induces human epithelial tumor cell migration and invasion via downregulation of E-cadherin. Acta Biochim Biophys Sin (Shanghai). 2016;48; 266-274.
[46] Guo Z, He Y, Wang S, Zhang A, Zhao P, Gao C, et al. Various effects of hepatoma-derived growth factor on cell growth, migration and invasion of breast cancer and prostate cancer cells. Oncology reports. 2011;26; 511-517.
[47] Moamer A, Hachim IY, Binothman N, Wang N, Lebrun JJ, Ali S. A role for kinesin-1 subunits KIF5B/KLC1 in regulating epithelial-mesenchymal plasticity in breast tumorigenesis. EBioMedicine. 2019;45; 92-107.
[48] Wang CC, Liau JY, Lu YS, Chen JW, Yao YT, Lien HC. Differential expression of moesin in breast cancers and its implication in epithelial-mesenchymal transition. Histopathology. 2012;61; 78-87.
[49] Schmit K, Michiels C. TMEM Proteins in Cancer: A Review. Front Pharmacol. 2018;9; 1345.
[50] Peng YG, Zhang ZQ, Chen YB, Huang JA. Rap2b promotes proliferation, migration, and invasion of lung cancer cells. Journal of receptor and signal transduction research. 2016;36; 459-464.
[51] Sung HY, Han J, Ju W, Ahn JH. Synaptotagmin-like protein 2 gene promotes the metastatic potential in ovarian cancer. Oncology reports. 2016;36; 535-541.
[52] Dimitrova Y, Gruber AJ, Mittal N, Ghosh S, Dimitriades B, Mathow D, et al. TFAP2A is a component of the ZEB1/2 network that regulates TGFB1-induced epithelial to mesenchymal transition. Biology direct. 2017;12; 8.
[53] Huang JJ, Corona AL, Dunn BP, Cai EM, Prakken JN, Blobe GC. Increased type III TGF-β receptor shedding decreases tumorigenesis through induction of epithelial-to-mesenchymal transition. Oncogene. 2019;38; 3402-3414.
[54] Liu X, Xu D, Liu Z, Li Y, Zhang C, Gong Y, et al. THBS1 facilitates colorectal liver metastasis through enhancing epithelial-mesenchymal transition. Clinical & translational oncology: official publication of the Federation of Spanish Oncology Societies and of the National Cancer Institute of Mexico. 2020;22; 1730-1740.
[55] Thorpe H, Asiri A, Akhlaq M, Ilyas M. Cten promotes epithelial-mesenchymal transition through the post-transcriptional stabilization of Snail. Mol Carcinog. 2017;56; 2601-2609.
[56] Lee KH, Ahn EJ, Oh SJ, Kim O, Joo YE, Bae JA, et al. KITENIN promotes glioma invasiveness and progression, associated with the induction of EMT and stemness markers. Oncotarget. 2015;6; 3240-3253.
[57] Liu TA, Jan YJ, Ko BS, Liang SM, Chen SC, Wang J, et al. 14-3-3ε overexpression contributes to epithelial-mesenchymal transition of hepatocellular carcinoma. PLoS ONE. 2013;8; e57968.
